# Low computed tomography coronary artery calcium scores in familial longevity: the Leiden Longevity Study

**DOI:** 10.1007/s11357-014-9668-6

**Published:** 2014-07-24

**Authors:** Lucia J. M. Kroft, Noortje van der Bijl, Jeroen van der Grond, Irmhild Altmann-Schneider, Pieternella E. Slagboom, Rudolf G. J. Westendorp, Albert de Roos, Antonius J. M. de Craen

**Affiliations:** 1 Department of Radiology, C2S, Leiden University Medical Centre, Albinusdreef 2, 2333 ZA Leiden, The Netherlands; 2 Netherlands Consortium for Healthy Ageing, Leiden, The Netherlands; 3 Department of Molecular Epidemiology, Leiden University Medical Centre, Leiden, The Netherlands; 4 Leyden Academy on Vitality and Ageing, Leiden, The Netherlands; 5 Department of Gerontology and Geriatrics, Leiden University Medical Centre, Leiden, The Netherlands

**Keywords:** Aging, Calcium score, Computed tomography, Longevity, Nonagenarian, Offspring

## Abstract

Offspring of long-lived parents have a low prevalence of cardiovascular disease in middle age. The purposes of this study were to investigate calcium scores in offspring as compared to controls and to determine the influence of cardiovascular risk factors. CT coronary artery calcium score was measured in offspring of long-lived families (*n* = 244, 125 males) and their partners (*n* = 223, 96 males) who served as controls. Calcium scores were analyzed separately for sexes. Subjects were grouped by very low calcium score ≤10 and scores above 10. Nonparametric Mann-Whitney test, chi-squared tests, and logistic regression analyses were performed to determine the association between calcium scores, familial longevity, and cardiovascular risk factors. More offspring of long-lived parents had lower calcium scores than controls. In men, 34 % of offspring had score ≤10 versus 21 % of controls (odds ratio (OR) and 95 % confidence interval (CI) 2.0, 1.08–3.7, *p* = 0.028). In women, 70 % of offspring had score ≤10 versus 54 % of controls (OR 1.9, 95 % CI 1.13–3.4, *p* = 0.019). Differences remained significant after correction for age (men, *p* = 0.043 and women, *p* = 0.003) and further correction for major risk factors in women, indicating genetic influence for lower calcium scores. In men, the association was found to be influenced by cardiovascular risk factors. Men and women with a familial propensity to become long-lived have lower coronary artery calcium scores than controls. Low scores may indicate a younger biologic arterial age associated with a low risk for incident cardiovascular disease.

## Introduction

Familial longevity is related to the low prevalence of cardiovascular disease and type 2 diabetes mellitus and is characterized by the preservation of insulin sensitivity and a beneficial lipid metabolism (Westendorp et al. 2009). The offspring of nonagenarian siblings, who are enriched for such advantageous familial influences on morbidity and mortality in comparison with their partners as age- and environment-matched controls, may serve as a good model to examine biological factors related to longevity (Westendorp et al. 2009).

The presence of coronary artery calcification as quantified by calcium scores is a direct marker for coronary atherosclerosis that can be assessed by computed tomography (CT). Calcium scores are widely used in clinical practice for cardiovascular risk stratification of asymptomatic subjects and patients (Greenland et al. 2007), since individual calcium scores are a strong predictor for myocardial infarction and cardiac death (Budoff et al. 2007; Greenland et al. 2004; Keelan et al. 2001; Kondos et al. 2003; Shaw et al. 2003; 2006a, b; Raggi et al. 2008; Vliegenthart et al. 2005). The most commonly used and validated scoring system for risk stratification is the Framingham risk score, which includes the major risk factors such as gender, total cholesterol, high-density lipoprotein cholesterol, systolic blood pressure or treatment of hypertension, cigarette smoking, and age (Greenland et al. 2007). CT-based calcium scores are associated with cardiovascular risk factors and provide direct evidence for coronary atherosclerosis. Large observational follow-up studies have shown that coronary calcium scoring provides incremental value over measured cardiovascular risk scoring in the prediction of mortality (Budoff et al. 2007; Greenland et al. 2004; Kondos et al. 2003; Shaw et al. 2003; Taylor et al. 2005). Furthermore, coronary calcium scores reflect the anatomic or biologic arterial age (Hoff et al. 2001; Kondos et al. 2003; Shaw et al. 2006a, b). Moreover, it has been shown that the biologic arterial age assessed by calcium score provides prognostic risk information incremental to chronological age and presence of risk factors such as hypercholesterolemia, hypertension, diabetes mellitus, and cigarette smoking (Kondos et al. 2003). It is not known whether individuals who have propensity to become long-lived have less coronary artery atherosclerosis.

The aims of this study were to investigate the calcium scores in the offspring of long-lived parents who have a familial propensity to become long-lived as compared to controls and to assess the relationship between cardiovascular risk factors and calcium scores.

## Materials and methods

### Patient selection

Subjects were included from the Leiden Longevity Study, which has been described in more detail elsewhere (Schoenmaker et al. 2006). In short, 421 Dutch Caucasian families were enrolled in the study between 2002 and 2006 based on the following inclusion criteria: (1) there were at least two living siblings per family, who fulfilled the age criteria and were willing to participate; (2) men had to be aged ≥89 years and women had to be aged ≥91 years; and (3) the sib pairs had to have the same parents. In 2002, only 0.5 % of Dutch men and 0.5 % of Dutch women were aged older than 89 and 91 years, respectively. The estimated probability that siblings within the Dutch population meet these age criteria is far less than 0.1 % (Westendorp et al. 2009). Additionally, offspring of these long-lived siblings were included as they have 35 % lower mortality rate compared to their respective birth cohorts in the general population. Their partners, who share the same socioeconomic and geographical background, were enrolled as environmentally and age-matched control group (Schoenmaker et al. 2006). For the current study, 447 subjects were recruited from the offspring of the long-lived siblings and their spouses, 223 offspring and 224 partners. The study was approved by our Institutional Review Board, and all participants gave informed consent.

### Cardiovascular risk factor assessment

At the baseline examination, demographic information and risk factors for coronary atherosclerosis were collected. Height, weight, and smoking status were obtained by self-reporting. History of myocardial infarction and diabetes mellitus was obtained from general practitioners. Hypertension was defined as use of antihypertensive medication as obtained from pharmacy records. The serum triglycerides, HDL cholesterol, and total cholesterol were measured from blood sampling.

### CT acquisition of calcium scores

Coronary atherosclerosis was evaluated by measurement of the Agatston total coronary artery calcium score using a 320-multidetector row CT scanner (Aquilion ONE, Toshiba, Otawara, Japan). The scan range was planned between the carina and cardiac apex. An unenhanced volumetric CT acquisition was performed with prospective ECG-triggering and 0.35-s CT tube rotation time. Imaging was performed within a single heartbeat during a breath-hold at inspiration. Tube voltage was 120 kV, and tube current varied between 200 and 400 mA, dependent on patient size and shape. Slice thickness was 0.5 mm, and reconstruction thickness 3.0 mm. Participants having coronary artery stents were not included for CT scanning for two reasons: (1) because image analysis was performed anonymized for clinical data, and stents preclude adequate image analysis; and (2) because of known stent(s), these participants automatically could be categorized to having the highest risk for coronary artery disease.

### Calcium score estimation and analysis

**Fig. 1:**
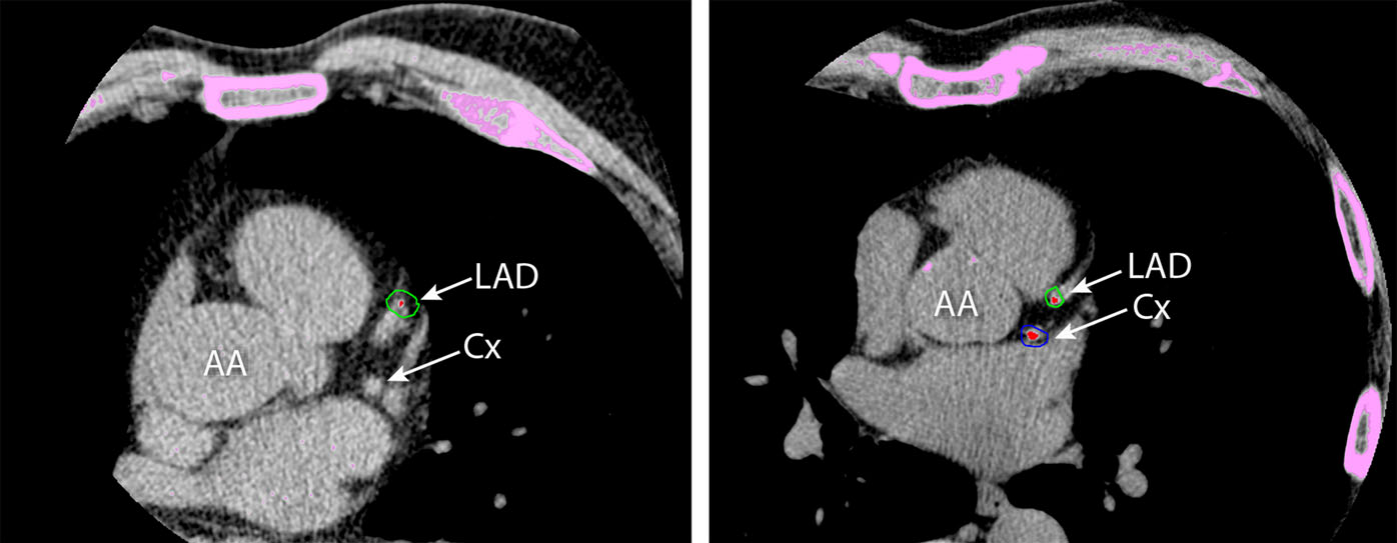
Computed tomography images in axial view showing coronary artery Agatston calcium scores on single slice levels. Total score is summed up for all slices. *Left*, offspring; 68-year-old male, height 1.79 m, weight 89 kg, no risk factors. Total Agatston calcium score was 3, by added scores of 0 in right coronary artery (RCA), 3 in left anterior descending artery (*LAD*), and 0 in circumflex artery (*Cx*). *Right*, control; 64-year-old male, height 1.77 m, weight 82 kg, with risk factor hypertension. Total Agatston calcium score was 68, by added scores of 5 in RCA, 19 in LAD, and 44 in Cx. *AA* ascending aorta, *S* sternum

Nonoverlapping 3.0-mm datasets were reconstructed, and images were transferred to a workstation for analysis (Vitrea FX, version 1.0, Vital Images). Dedicated CT calcium score analysis software (VScore, Vital Images) was used. Pixels exceeding the threshold value of 130 HU were automatically recognized by the postprocessing tool. These areas were manually encircled in the course of the coronary arteries. The amount of coronary artery calcification was automatically calculated according to Agatston et al. (1990). The Agatston score is a weighted score dependent on peak attenuation in Hounsfield units and a calcium area, summed up for all slices in the volume covering the coronary arteries (Agatston et al. 1990). Calcium score was expressed as Agatston total score (Fig. 1). Scores were categorized into very low calcium score (≤10) and low-to-extensive calcium score (>10). This was done because very low scores (0–10) identify subjects with very low risk for coronary artery disease, and low-to-extensive calcium scores (>10) identify subjects with increasing severity grades reflecting increasing likelihood of coronary artery disease (Bellasi et al. 2007; Blaha et al. 2009; Budoff et al. 2007; Geluk et al. 2008; Raggi et al. 2004; Shaw et al. 2003; 2006a, b). In addition, the threshold of calcium scores 0–10 versus >10 was chosen as this seems the most discriminating by means of risk for mortality. Because of the effects on risk, we therefore combined calcium scores 0 and 1–10 versus higher scores. For overview of calcium score distribution, participants were also categorized according to Rumberger: zero score (no measurable calcified plaque), 1–10 minimal, 11–100 mild, 101–400 moderate, and >400 extensive calcified plaque (Rumberger et al. 1999). All imaging data were analyzed blinded, both for group (offspring or partner) and clinical information.

### Statistical analysis

Calcium scoring grades are highly related to age and sex (Hoff et al. 2001). Because the proportion of male and female participants was not equal in the offspring and control groups, calcium score data was analyzed separately for men and women.

Categorical baseline data are expressed in numbers and percentages. The Mann-Whitney test was used for continuous variables and chi-squared test for dichotomous variables. Continuous variables are expressed as mean value ± standard deviation and/or median values with a 25–75th percentile (interquartile range (IQR)). Calcium scores for men and women were calculated per quartile age categories. Eleven participants with known stents were not included in numeric calcium score calculations but were included in the group with calcium score >10 or according to Rumberger in group >400.

Differences between offspring enriched for familial longevity and controls were compared with logistic regression analysis. Univariate logistic regression was performed to calculate odds ratios with corresponding 95 % confidence intervals (95 % CIs). Multiple variable logistic regression was performed to identify parameters that might influence the association between familial longevity and coronary calcification, including age, diabetes mellitus, hypertension, body mass index, lipids, and current smoking. Statistical analysis was performed using SPSS software (version 20.0 SPSS Inc., Chicago, IL, USA). A *p* value of <0.05 was considered statistically significant.

## Results

### Study population

The clinical and demographic characteristics of the 447 participants are given in Table 1. Among men, there were 125 offspring and 96 controls, while among women, there were 99 offspring and 127 controls. In men, diabetes mellitus (3.4 vs 12.6 %, *p* = 0.013) and hypertension (17.6 vs 31.3 %, *p* = 0.018) were less frequent in offspring than in controls.

**Table 1:** Subject characteristics

	Men			Women		
	Offspring (*n* = 125)	Controls (*n* = 96)	*p* value	Offspring (*n* = 99)	Controls (*n* = 127)	*p* value
Age (years, mean ± SD)	66.6 ± 6.1	67.5 ± 6.9	0.29	65.4 ± 5.8	64.0 ± 6.8	0.09
BMI (kg/m^2^, mean ± SD)	27.0 ± 3.0	26.7 ± 3.1	0.50	25.7 ± 4.6	26.6 ± 4.4	0.13
Diabetes mellitus (%)	4 (3.4)	11 (12.6)	0.013	2 (2.4)	7 (6.0)	0.21
Current smoker (%)	16 (12.8)	13 (13.5)	0.87	10 (10.1)	17 (13.4)	0.46
Myocardial infarction (%)	2 (1.7)	4 (4.8)	0.21	0 (0)	1 (0.9)	0.38
Hypertension (%)	22 (17.6)	30 (31.3)	0.018	21 (21.2)	27 (21.3)	0.98
Triglycerides (mmol/L)	0.60 ± 0.55	0.57 ± 0.51	0.71	0.30 ± 0.52	0.35 ± 0.54	0.46
HDL cholesterol (mmol/L)	1.30 ± 0.39	1.25 ± 0.33	0.30	1.65 ± 0.46	1.58 ± 0.56	0.25
Total cholesterol (mmol/L)	5.57 ± 1.17	5.57 ± 1.10	0.99	5.70 ± 1.35	5.71 ± 1.19	0.98

### CT calcium score

**Fig. 2:**
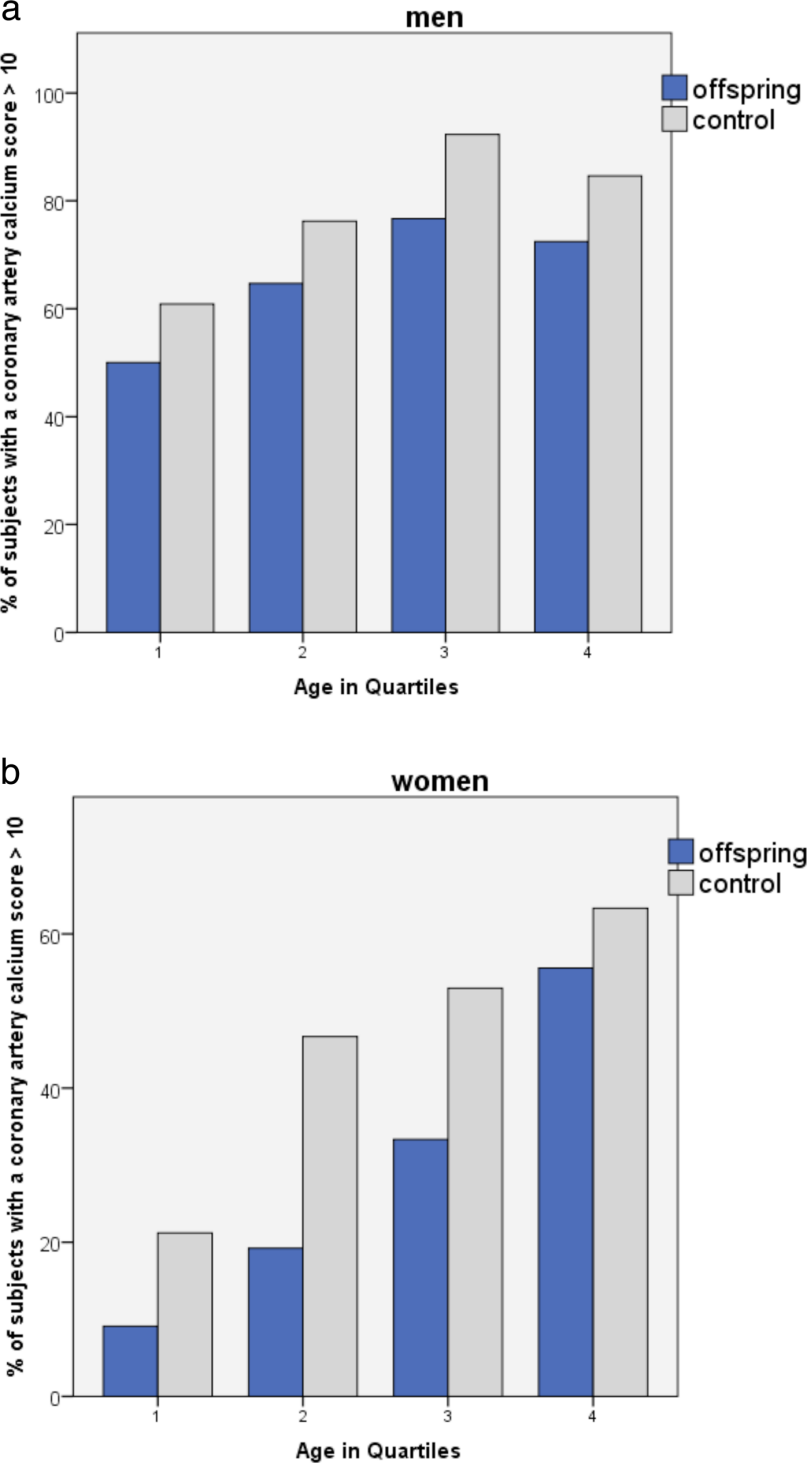
The percentage of subjects for men (**a**) and women (**b**) with calcium scores >10 for quartile age categories within each group. Age groups were divided by comparable numbers of 55–58 subjects in each quartile group. Quartile mean ages for groups in men (**a**) are as follows: group 1 aged 48.7–62.8 years, mean age 58.8 years; group 2 aged 62.9–67.1 years, mean age 64.9 years; group 3 aged 67.2–71.1 years, mean age 69.0 years; and group 4 aged 71.2–84.4 years, mean age 75.2 years. Quartile mean ages for groups in women (**b**) are as follows: group 1 aged 45.5–61.0 years, mean age 56.3 years; group 2 aged 61.1–64.9 years, mean age 62.8 years; group 3 aged 65.0–68.8 years, mean age 66.9 years; and group 4 aged 68.9–81.6 years, mean age 72.4 years. In all age groups, for men and women, the percentage individuals with calcium score >10 was lower for offspring of long-lived parents than for controls. Also, the percentage calcium scores >10 increase with age. The figures suggest that offspring are biologically younger for their age as compared to age-matched controls

Figure 1 shows an example of CT calcium scoring in offspring and control. In men, median (IQR) calcium score in offspring was 58 (1–295) versus 79 (14–440) in controls (*p* = 0.08). In women, median (IQR) calcium score in offspring was 0 (0–25) versus 3 (0–75) in controls (*p* = 0.09). Whereas the median calcium score did not differ between the groups, the distribution was significantly different. Table 2 shows the distribution of the calcium scores ≤10 versus >10 for men and women. Significantly, more male offspring from long-lived parents had a calcium score ≤10 than male controls; 34 % of offspring had a calcium score ≤10 versus 21 % of controls (odds ratio (OR) = 2.0, 95 % CI 1.08–3.7, *p* = 0.028). In females, 70 % of offspring had a calcium score ≤10 versus 54 % of controls (OR = 1.9, 95 % CI 1.1–3.4, *p* = 0.019). Figure 2 shows for men and women the percentage of individuals with calcium scores >10 for quartile age categories with comparable numbers of participants within the groups. In all age groups, for both men and women, the percentage of individuals with calcium score >10 was lower for the offspring of long-lived parents than for controls. In addition, the percentage calcium score >10 increases with age. Figure 2 suggests that offspring are biologically younger for their age as compared to age-matched controls.

**Table 2:** Distribution of Agatston calcium score (≤10 vs >10) in offspring and controls, stratified by sex

	Men				Women			
	Offspring (*n* = 125)	Controls (*n* = 96)	OR (95 % CI)	*p* value	Offspring (*n* = 99)	Controls (*n* = 127)	OR (95 % CI)	*p* value
Calcium score (*n*, %)								
< 10	43 (34)	20 (21)	1 (ref)		69 (70)	69 (54)	1 (ref)	
> 10	82 (66)	76 (79)	2.0 (1.08–3.7)	0.028	30 (30)	58 (46)	1.9 (1.1–3.4)	0.019

Table 3 shows the calcium scores by stratification according to Rumberger et al. (1999). In men, the offspring revealed significantly lower calcium score risk groups than controls (*p* = 0.016). Because of the overall small groups and overall lower calcium scores in women than in men, differences between those multiple categories did not reach statistical significance in women, although a trend (*p* = 0.130) towards lower category scores for offspring was observed in women as well.

**Table 3:** Agatston calcium score groups according to Rumberger

		CS 0	CS 1–10	CS 11–100	CS 101–400	CS >400	*p* value
Men	Offspring (%)	28 (22)	15 (12)	28 (22)	32 (26)	22 (18)	0.016*
	Controls (%)	11 (11)	9 (9)	29 (30)	16 (17)	31 (32)	
Women	Offspring (%)	53 (54)	16 (16)	14 (14)	9 (9)	7 (7)	0.130
	Controls (%)	57 (45)	12 (9)	32 (25)	17 (13)	9 (7)	

*CS* chi-squared test (cross tabulation)

*p < 0.05

Table 4 shows the results of the multiple variable logistic regression analysis to identify which parameters influence the observed association between offspring and controls. Adjustment for age did not affect the association in both men (OR 1.9, *p* = 0.043) and women (OR 2.5, *p* = 0.003). After modeling with adding corrections for diabetes mellitus and hypertension, and additionally for body mass index, lipids, and current smoking, the difference in calcium score between offspring and controls was still significant in women (OR 2.4, *p* = 0.016) but attenuated in men (OR 1.4, *p* = 0.38).

**Table 4:** Distribution of Agatston calcium score (≤10 vs >10) in offspring and controls, stratified by sex

	Men			Women		
	Odds ratio	95 % CI	*p* value	Odds ratio	95 % CI	*p* value
Crude	2.0	1.08–3.7	0.028	1.9	1.13–3.4	0.019
Model I	1.9	1.02–3.6	0.043	2.5	1.36–4.6	0.003
Model II	1.5	0.78–2.9	0.23	2.6	1.35–5.1	0.004
Model III	1.4	0.68–2.8	0.38	2.4	1.18–4.7	0.016

*Model I* adjusted for age; *Model II* adjusted for age, diabetes mellitus, and hypertension; *Model III* adjusted for age, diabetes mellitus, hypertension, body mass index, triglycerides, HDL cholesterol, total cholesterol, and current smoking

## Discussion

The major finding of our study is that offspring of long-lived families have more frequently low calcium scores than age-matched control subjects and that this difference is observed in both sexes independent of age. In women, such lower calcium scores were even independent of presence of concomitant diabetes mellitus, hypertension, body mass index, lipids, and smoking. These findings suggest that offspring of long-lived parents with a familial propensity to become long-lived are biologically healthier than their age-matched controls as estimated from the coronary artery calcium score.

We distinguished groups of subjects for risk based on very low calcium score ≤10 versus scores above 10, as previous studies have demonstrated a fundamental difference in risk between these scores regarding prognostic risk for mortality. In a large population study (44,052 patients), the mortality risk was four times higher in those with scores above 10 as compared to those with scores below 10 (Blaha et al. 2009). In another study comprising 10,377 asymptomatic patients in which 57 % had scores of 10 or less, survival after a 5-year follow-up was 99.0 % for those with calcium scores of 10 or less. Relative risk of mortality was 1.64 to four times greater in those who had varying degrees of calcium scores above 10 (Shaw et al. 2003). Furthermore, a meta-analysis with pooled data of 55,807 patients reported all-cause annual mortality rates of 0.1 % for women and men with calcium scores up to 10 versus 1.6 % for women and 2.6 % for men with calcium scores above 10 (Bellasi et al. 2007). In another study comprising 35,388 asymptomatic patients, hazard ratio for death was found highly dependent on calcium score and age. In the age group 60–69 years (3,519 women and 3,926 men, age comparable to our study group), hazard ratio increased from 2.1 to 5.1 for scores above 10 (Raggi et al. 2008). These studies demonstrate the clinical relevance of very low calcium scores in relationship with survival. In accordance with these previous observations, our study revealed more often a very low calcium score ≤10 in the offspring of long-lived parents as compared to control subjects indicating that their risk for cardiovascular disease is substantially lower. In addition, we observed differences between men and women, by showing that women had lower calcium scores than men. The dependency of calcium scores on age and sex is well known (Hoff et al. 2001). Between sexes, calcium scores have shown equally related to predicting obstructive coronary artery disease (Budoff et al. 2002), although for comparable age groups and calcium scores, survival has shown better for women than for men (Raggi et al. 2008). It has been stated that regarding calcium scores, women chronologically lag 14 to 15 years of age behind men (Budoff et al. 2002; Hoff et al. 2001).

The age dependency of calcium scores has lead to the concept of calcium score as indicator of biologic arterial aging (Hoff et al. 2001; Shaw et al. 2006a, b). Indeed, increasing coronary calcium has shown predictive of increasing mortality for all age groups, and elderly people without calcium have a lower mortality rate than younger persons with high calcium scores (Tota-Maharaj et al. 2012). It has been proposed to adjust patients’ age on the basis of the gender-specific level of atherosclerosis by calcium score, as to provide patients with a more understandable version of their calcium scores (e.g., “you are 55 years old but your arteries are more consistent with an arterial age of 65 years”). Moreover, the biologic arterial age as function of calcium score was found more predictive of short-term incident coronary events than Framingham risk based on observed age (McClelland et al. 2009; Shaw et al. 2006a, b). Also, in subjects older than 70 years, calcium scores ≤10 can be interpreted as having a calendar age minus 10 years (Shaw et al. 2006a, b). Accordingly, in offspring of long-lived families, lower calcium scores may be interpreted as younger arterial age as compared to the control group. We found for both men and women that the offspring of long-lived families stratified by age group had more often low calcium scores suggesting younger biologic age as compared with controls. Previous investigations of the Leiden Longevity Study have shown that the offspring of long-living elders are genetically enriched for extreme survival (Schoenmaker et al. 2006). Therefore, the offspring of these long-living parents are likely genetically protected against arterial aging.

In men, the relationship between familial longevity and low calcium scores attenuated after correction for cardiovascular risk factors, while diabetes mellitus and hypertension were more present in controls than in long-lived offspring. Therefore, in males, the difference between offspring and controls in prevalence of low calcium scores ≤10 is mainly explained by differences in prevalence of diabetes and hypertension between the groups. Previous studies have indeed shown lower prevalence of myocardial infarction, hypertension, and diabetes mellitus in offspring of familial nonagenarians (Westendorp et al. 2009) and beneficial metabolic profiles. It is therefore likely that long-lived offspring are protected against development of coronary calcium deposition by beneficial metabolic profiles. Moreover, in women, with overall lower prevalence of diabetes mellitus and hypertension, lower calcium scores for long-lived offspring as compared to controls remained after correction for major risk factors diabetes mellitus and hypertension and also after additional correction for risk factors body mass index, lipids, and smoking. These findings further indicate that even with comparable metabolic profiles, calcium scores are lower in offspring of long-lived families than in controls, suggesting additional genetic influence protecting against arterial aging with less coronary artery atherosclerosis in offspring of long-lived families. Deceleration of arterial aging may go hand in hand with the known remarkable metabolic health of these families and explain their long life span.

### Study limitations

Offspring of long-lived families were environmentally matched by using their partners as controls. Because of known differences in calcium scores between men and women, analyses were separately performed for sexes. As distribution of men and women within the groups was not equal, male offspring was compared to male partners and female offspring to female partners. Therefore, individual participants were not pair-wise matched with their partners, although the overall study population was environmentally and age matched, which is unique in design.

In conclusion, men and women with familial propensity to become long-lived have lower coronary artery calcium scores than age-matched controls. In multiple studies, low scores that may indicate a younger biologic arterial age have been associated with lower risk for incident cardiovascular disease. Lower biologic arterial age may contribute to health and a longer life span in familial longevity.
